# Bacterial composition and function during fermentation of Mongolia koumiss

**DOI:** 10.1002/fsn3.2377

**Published:** 2021-06-12

**Authors:** Yue Wu, Yu Li, Qimu Gesudu, Juntao Zhang, Zhihong Sun, Haobisi Halatu, Bilege Menghe, Wenjun Liu

**Affiliations:** ^1^ Key Laboratory of Dairy Biotechnology and Engineering Ministry of Education of China Inner Mongolia Agricultural University Hohhot China; ^2^ Key Laboratory of Dairy Products Processing Ministry of Agriculture and Rural Affairs of China Inner Mongolia Agricultural University Hohhot China; ^3^ Inner Mongolia Key Laboratory of Dairy Biotechnology and Engineering Inner Mongolia Agricultural University Hohhot China; ^4^ Inner Mongolia International Mongolian Hospital Hohhot China

**Keywords:** bacterial function, koumiss, metagenomics, nutritional composition, pathways

## Abstract

Koumiss is a fermented mare's milk beverage that has attracted increasing attention due to its nutritional richness and important economic value. Bacteria in koumiss play a major role in pH decreasing and reducing spoilage through bacterial inhibition. The dynamic changes in nutritional content were determined firstly during fermentation, and then the metagenomics sequencing technology was applied to profile koumiss core microbiota at the species level. We also clarified the function and effect of the bacteria on the nutritional content of the final product. We also investigated active microbial function by comparing the metagenomics of representative samples collected at different time points during the fermentation process. This study dynamically revealed the bacterial composition and function of traditional koumiss during its making process. Twenty‐three major functional categories related to amino acid and fat synthesis, metabolism, and so on were identified. Functional category L (represented replication‐, recombination‐, and repair‐related functions) was one of the most important categories with the highest relative abundance in all of the 23 major functional categories. CoG category having a significant correlation with *Lactococcus piscium* was the most abundant. The change in metabolic activity of bacteria at different fermentation time points showed that the metabolic activity was more active in the first 24 hr and then began to stabilize. LAB play the major role in the koumiss pH decreasing and quality improvement. The functional genes of related metabolic activity of lactic acid bacteria were more active in the first 24 hr of koumiss fermentation and then began to stabilize.

## Graphical abstract

The main functional categories that can participate in metabolism, etc. The change of metabolic activity of bacteria at different fermentation time points.

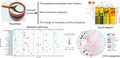

## 1 INTRODUCTION

Koumiss, also called Airag, or Chige is a spontaneously fermented mares’ milk beverage that is very popular in Central Asia; it is thought to be of Huno‐Bulgar, Turkic and Mongol origin and has been drunk habitually since ancient times (Sudun et al., 2013). Koumiss can be mildly alcoholic, but would need to be consumed in enormous quantities to have any noticeable effects (Bakir et al., 2015; Jagielski, 1874). It is produced using traditional method sandan indigenous starter culture (i.e., previously made koumiss) containing lactic acid bacteria (LAB), yeast, and other organisms associated with fermentation (Mu et al., 2012; Sun, Liu, Zhang, Yu, Gao, et al., 2010; Watanabe et al., 2008; Wu et al., 2009). However, the literature concerning the dynamic changes in bacterial composition and function with respect to the nutrition of ancient koumiss is scarce.

The nutritional components of koumiss are very rich and include fat, protein, vitamins, amino acids, carbohydrates, and trace mineral elements (Zhang & Zhang, 2012). Traditionally, it was considered a functional food, not only providing rich nutrients, but also due to its medicinal properties. It is considered to be beneficial in postoperative care (Jagielski, 1877; Thompson, 1879). The Cossacks have introduced koumiss into military rations to prevent tuberculosis (Ishii & Samejima, 2001). Chen et al. (Chen et al., 2010) found that koumiss was rich in angiotensin I‐converting enzyme inhibitory peptides, which have antihypertensive properties. Chaves‐López et al. (Chaves‐López et al., 2012) also reported angiotensin I‐converting enzyme inhibitory activity in yeast strains isolated from Colombian koumiss. But, at present, it is just as functional as any other fermented dairy product (including all cheeses). Despite this, few studies have clarified the interactions among microorganisms and describe the detailed function of these bacteria with respect to metabolite formation using metagenomics analysis. Restrictions associated with these techniques have left the scientific community with a limited view of the total diversity and functionality present in complex bacterial communities. New next‐generation sequencing (NGS) technologies are a powerful tool to explore microbial function globally in food samples, including genes involved in microbial metabolism, and to characterize microbial composition using marker genes. Shotgun metagenomic analysis methods can infer both taxonomic and functional information in complex microbial communities. This culture‐independent method can improve the existing knowledge about microbial community structure and function by enabling direct identification of genetic material from environmental samples (Almeida et al., 2019). In the present study, nutrient composition and bacterial biodiversity were evaluated at different time points during koumiss fermentation and storage. The function of bacteria during koumiss fermentation was elucidated using metagenomic sequencing.

## 2 MATERIAL AND METHODS

### 2.1 Samples collection

A total of six samples were taken, over time, from traditionally fermented koumiss from one site in Xilingol, Inner Mongolia, which is located at latitude 43.94 and longitude 116.05. These samples (B_1_, B_2_, B_3_, B_4_, B_5_, and B_6_) were taken at different stages of fermentation (0, 12, 24, 36, 48, and 168 hr). They were collected aseptically and were kept in a tank filled with liquid nitrogen during transportation (Liu et al., 2019).

### 2.2 Biochemical analysis

The fat content of koumiss samples was determined using the Rose Gottlieb method (Nos. 991.20, 905.02, respectively) described by the Association of Official Analytical Chemists (AOAC 1997). The total protein content was determined using the Kjeldahl nitrogen method and calculated from total nitrogen using a 6.38 conversion factor (Kirk, 1950).

Lactic acid and fatty acid contents were determined using HPLC (Liu et al., 2019). Lactose content was determined using the FIL‐IDF 147B method (FIL‐IDF Standard 1998). Pretreatment of samples followed the methods described by Choi (2016). The HPLC system (Varian) consisted of a solvent delivery system (9012Q), a refractive index detector (Star 9,040), and a column oven (101). The temperature of the 150 × 7.7 mm Rezex ROA column (Phenomenex) was 60℃. The eluant was 4 mM sulfuric acid and the flow rate was 0.6 ml/min.

### 2.3 Nutritional composition testing

#### 2.3.1 Determination of vitamin composition

Levels of water‐soluble vitamin B_1_ (VB_1_) and B_2_(VB_2_) were determined using HPLC based on the methods of Zafra‐Gómez et al. (Zafra‐Gómez et al., 2006). Separation of vitamin E was done using reverse‐phase liquid chromatography (RP‐LC) according to the methods of Gámiz‐Gracia et al. (Gámiz‐Gracia et al., 1999) and modified for the samples collected. After saponification, 50 ml sample was extracted by petroleum ether and prepared before HPLC analysis according to GB/T 5,009.82–2003 methods (AOAC 1997). Then 20 µl of the extract was analyzed by RP‐LC (Liu et al., 2019).

#### 2.3.2 Quantification of amino acids

The quantity of 17 common amino acids in the samples of koumiss taken at different stages of fermentation was measured in order to determine changes in their content. Details of the method used are described in Liu et al. (Liu et al., 2019). Briefly, 1 ml from each koumiss sample was placed into special glass tubes. 10 ml 6 M HCl was added to each tube, and then the tubes were sealed after vacuum treatment. Tubes were placed in dry boxes at a constant temperature of 110℃ to hydrolyze for 24 hr, after which time they were allowed to cool. Then, the hydrolysate was diluted with distilled water to a constant volume of 50 ml; 1 ml of this solution was dried in a vacuum and ground into a powder. Each of the samples was then homogenized in 1.2 ml of 0.02 M HCl and filtered through a 0.22 μm mesh to obtain the final solution. The amino acid content was measured using a Hitachi L‐8800 automatic amino acid analysis unit with an ion exchange resin separation column that had a diameter of 4.6 mm ×60 mm (ion exchange resin 2622SC). The temperature of the separation column was set at 57℃. The flow speed of the buffer solution and ninhydrin was 0.4 ml/min (pressure 7.0–8.5 MPa) and 0.35 ml/min (pressure 0.9–1.1 MPa), respectively. After acid hydrolysis, the amino acids present in the koumiss were analyzed using an amino acid analyzer (L‐8900, HITACHI, Japan).

#### 2.3.3 Genomic DNA extraction

Whole genomic DNA was extracted using the methods of Gesudu et al. (Gesudu et al., 2016). The quality of extracted DNA was checked by 0.8% agarose gel electrophoresis and spectrophotometry (optical density at 260 nm/280 nm ratio) with a micro‐ultraviolet spectrophotometer to determine its concentration. All extracted DNA samples were stored at −20℃ before subsequent analysis.

#### 2.3.4 Metagenomic sequencing

All samples were sequenced using an Illumina HiSeq 2 000 instrument, by Shanghai Major Biobío‐pharm Technology Co., Ltd. Sequencing libraries were prepared with a fragment length of approximately 300 bp. Paired‐end reads were generated with 100 bp in the forward and reverse directions. The sample taxonomic profile was also determined directly from the metagenomic dataset using two online software packages, Parallel‐META (Su et al., 2014) and MetaPhlAn (Segata et al., 2012), following the instructions of the two software developers.

#### 2.3.5 Functional annotation

Putative functional genes and amino acid sequences predicted in our gene catalog were aligned with the proteins/domains in the Cluster of Orthologous Group (COG) and Kyoto Encyclopedia of Genes and Genomes (KEGG) databases using BLASTP (e‐value ≤6 1e‐5 with a bit‐score higher than 60). Each protein was assigned to the Cluster of Orthologous Groups (COGs) (Tatusov et al., 2001) or Kyoto Encyclopedia of Genes and Genomes (KEGG) orthologues group (KOs) (Kanehisa & Goto, 2000) by the highest scoring annotated hit.

#### 2.3.6 Variation in metabolic pathways at different stages of fermentation based on KEGG

In order to identify changes in metabolic activity of each pathway at different fermentation time points, changes in metabolic activity of each pathway were compared at two adjacent time points. Pathways satisfying the following conditions were considered to be different between two adjacent time points: (a) KO detected in one pathway was more than 50% of the total KOs in the pathway; (b) There was at least 80% or 90% (difference threshold) of KO in which relative content in one sample was more than that in another.

### 2.4 Statistical analyses

Statistical analyses were performed mainly using the R packages, including ade4 ggplot2, (http://www.r‐project.org/), together with Python (Sanner, 1999), Canoco for Windows 4.5 (Microcomputer Power), and PAST (Hammer et al., 2001). Differences in the relative abundances of taxonomic groups between samples at gene level were evaluated using a Mann–Whitney test. False discovery rate (FDR) values were estimated using the Benjamini–Yekutieli method to control for multiple testing (Benjamini & Yekutieli, 2001). *p*‐values <.05 were considered statistically significant. Differentially expressed genes were identified using DESeq2 (Love et al., 2014).

## 3 RESULTS

### 3.1 Nutritional composition and content

The content of lactose in fresh mares’ milk (0 hr) was 45.75 ± 2.15 mg/L. The content of lactic acid in koumiss ranged from 8.2 ± 1.21 to 32.13 ± 1.54 mg/L and began increasing as soon as the fresh mares’ milk was seeded with the starter culture from aged koumiss at the beginning of fermentation. The protein content of fresh mares’ milk was 1.86 ± 0.07%, and the koumiss was 2.22 ± 0.08%; there was no significant difference between fresh milk and fermented milk. Fat content ranged from 0.74 ± 0.16% to 1.48 ± 0.08%, and there was no significant difference between fresh milk and fermented milk. VB_1_ and VB_2_ concentration ranged from 1.48 ± 0.12 to 4.86 ± 0.42 µg/100 g and from 2.48 ± 0.12 µg/100 g to 4.86 ± 0.32 µg/100 g, respectively, and there was no significant difference between fresh milk and fermented milk. Vitamin E (VE) concentration ranged from 44.63 ± 0.63 to 99.90 ± 0.05 µg/100 g, and there was no significant difference between fresh milk and fermented milk. The content of the 17 amino acid ranged from 2.40 ± 0.11 µg/100 g for methionine (MET) to 39.64 ± 0.01 µg/100 g for glutamate (GLU). There was significant variation in the content of these amino acids which increases significantly as fermentation time increased. However, the content of cysteine (CYS) and histidine (HIS) decreased gradually with increased fermentation time. The results are shown in Table. 1.

**TABLE 1:** Comparison of nutrient composition between fresh mares’ milk and koumiss

Nutrients	Fresh mares’ milk	Koumiss
Lactose (mg/L)	45.75 ± 2.15	46.12 ± 1.93
Lactic acid (mg/L)	8.2 ± 1.21 ~ 32.13 ± 1.54	
Protein (%)	1.86 ± 0.07	2.22 ± 0.08
Fat (%)	0.74 ± 0.16 ~ 1.48 ± 0.08	
VB1 (µg/100 g)	1.48 ± 0.12 ~ 4.86 ± 0.42	
VB2(µg/100 g)	2.48 ± 0.12 ~ 4.86 ± 0.32	
Vitamin E (µg/100 g)	44.63 ± 0.63 ~ 99.90 ± 0.05	

### 3.2 Microbial diversify in the koumiss at different stages of fermentation

**FIGURE 1:**
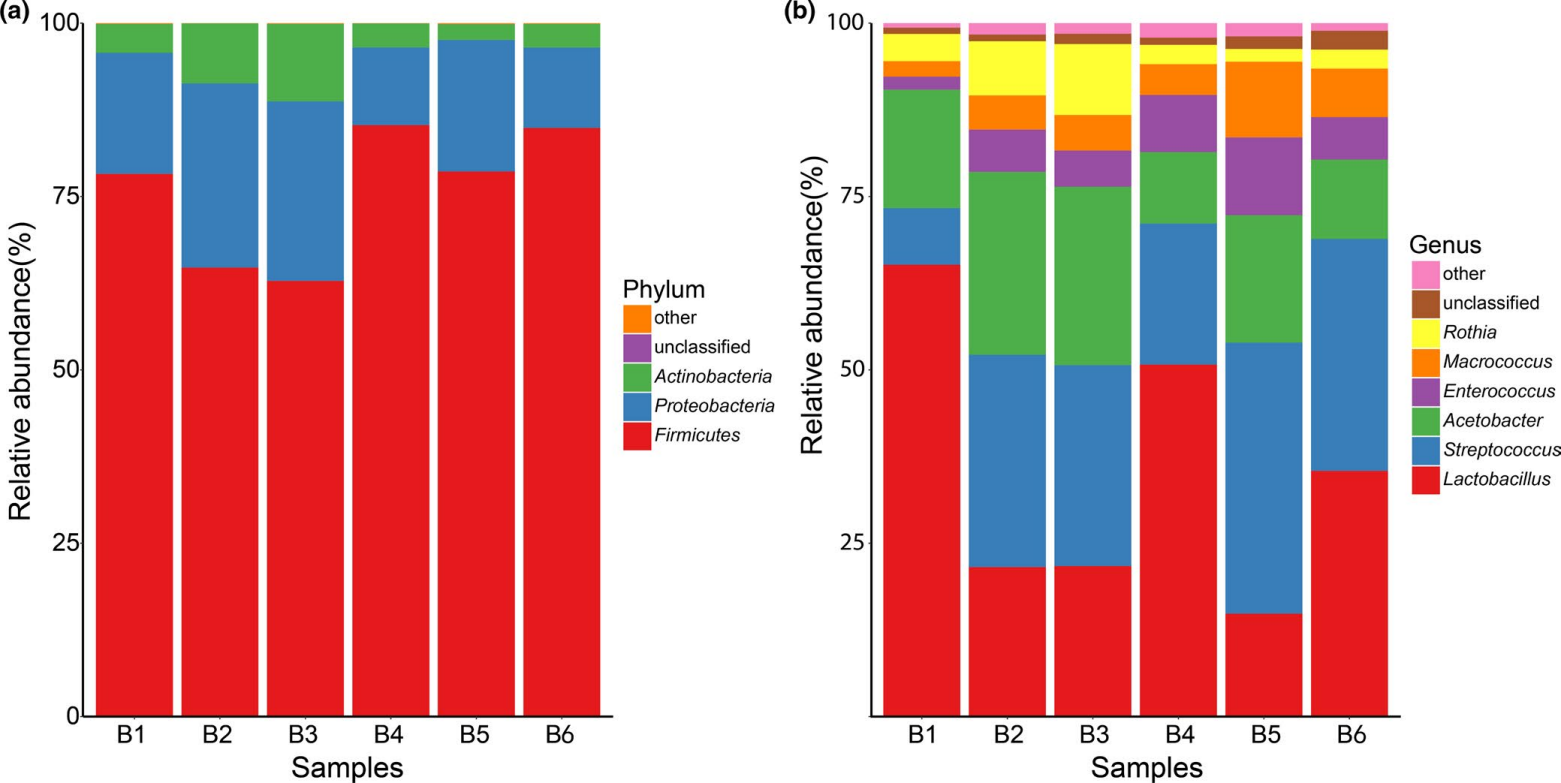
Relative abundance of bacteria diversity detected in different koumiss samples. Note: (a) and (b) means respectively relative abundance of bacteria based on Phylum and genus

Changes in bacteria diversify of koumiss during fermentation are presented in Figure 1. The genus of *Lactobacillus* in the phylum Firmicutes was the predominant bacterial genus throughout fermentation. However, with the extension of fermentation time, *Lactobacillus* tended to decrease while *Streptococcus* tended to increase in abundance.

### 3.3 Changes in microbial functional genes during fermentation

Following trimming and filtering, whole genome shotgun sequencing of six samples achieved 111.70 Gb (18.62 ± 2.09 Gb) data. After assembling, 495,259 contigs were obtained. The average N50 length of the contigs was 3,127 bp. A total of 858,433 genes were obtained after MetaGeneMark prediction, and these results are summarized in Table. 2.

**TABLE 2:** Genome sequencing results of each group samples

Samples		Raw sequencing data		Single sample sequence assembly				Predicted gene	
	Read length (bp)	Numbers of reads (million)	Data of each sample (Gb)	Contig	Number of bases (million)	N50 length(bp)	Average read length (bp)	Number of predicted genes	Average read length (bp)
B_1_	101	59.73	18.6	47,275	69.78	4,587	1,476.14	93,172	610.16
B_2_	101	61.17	19.5	97,835	111.14	2,673	1,135.95	160,354	547.59
B_3_	101	50.47	15.7	73,798	93.16	3,296	1,262.30	132,823	570.76
B_4_	101	65.91	22	100,349	107.60	2,288	1,072.29	158,345	533.44
B_5_	101	55.43	17.6	80,569	102.93	3,138	1,277.59	147,041	572.28
B_6_	101	57.48	18.3	95,433	114.97	2,780	1,204.72	166,698	558.43

Note

Contig represented the long fragments formed after assembly of short sequences; N50 can be described as a weighted median statistic such that 50% of the entire assembly is contained in contigs or scaffolds equal to or larger than this value.

The entire sequence data set has been deposited in the National Center for Biotechnology Information (NCBI) Sequence Read Archive (SRA) database (accession number: PRJNA527179).

### 3.4 Identification of functional categories based on COG databases

**FIGURE 2:**
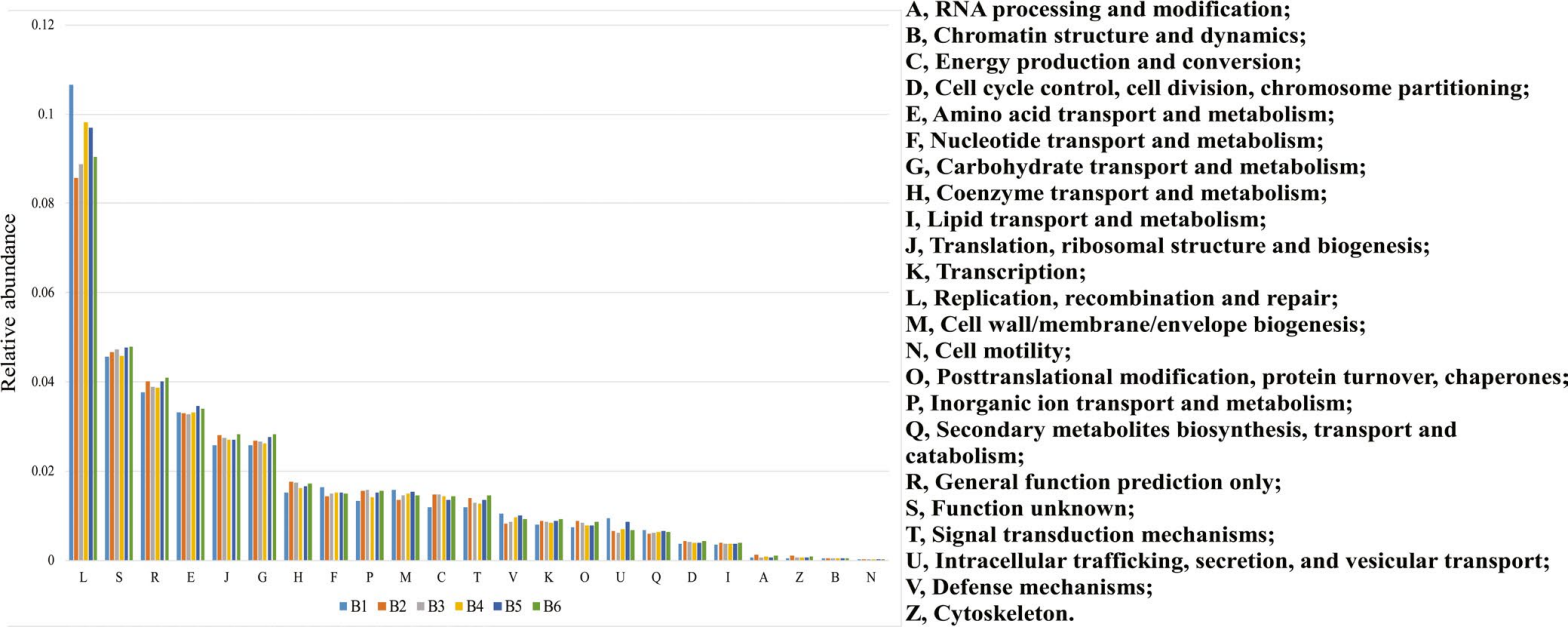
Relative abundance of main COG functional categories of each sample group

The protein function and metabolic pathway annotation information were obtained from BLAST analysis by searching the protein sequences in the COG database, which are translated from genes. A total of 1883 COG functional units were identified from the six samples in this study. These sequences represented 42.83% of all the sequences that could be aligned to the COG database. These functional units fell into 23 major functional categories, and the relative content of the major functional categories in each sample is shown in Figure 2.

From Figure 2 We can determine that functional category L (representing replication‐, recombination‐, and repair‐related functions) is one of the most important of the 23 major functional categories with the highest relative abundance. Function category S is the second largest category, and the function category is unknown. Functional category R (representing general functions) was the third largest category. In addition, functional categories E (amino acid transport, metabolism), J (translation, ribosome structure, and biosynthesis), and G (carbohydrate transport and metabolism) had a relatively high abundance in these six samples.

### 3.5 Bacteria function in relation to nutrition and chemical composition of fermented milk

**FIGURE 3:**
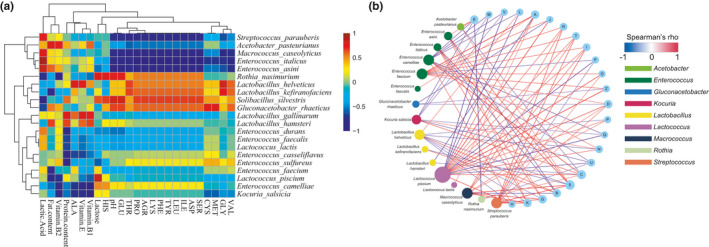
Relationship between chemical and nutrition content and bacterial species (a), and correlation analysis between COG categories and predominant bacterial species of koumiss (b)

The relationships between bacterial species and nutritional composition as well as the content of fermented milk were identified (Figure 3a). Lactic acid content was significantly positively correlated with *S*. *parauberis*, *Macrococcus caseolyticus*, *E. italicus,* and *E. asini* (*p* < .05). Fat content was positively correlated with *Acetobacter pasteurianus* and *Lactobacillus hamster* (*p* < .05), but negatively correlated with *E. faecium* and *Lc.piscium* (*p* < .05). VB_2_ was positively correlated with *A*. *pasteuriamus* and *Gluconacetobacter rhaeticus* (*p* < .05). Protein content was significantly positively correlated with *L. gallinarum* and *Lb.hamsteri* (*p* < .05), but significantly negatively correlated with *E. sulfureus* and *E. casseliflavus* (*p* < .05). Alanine, VE, and VB_1_ were positively correlated with *L. helveticus* and *Lb.gallinarum* (*p* < .05), but negatively correlated with *E. casseliflavus*, *E. camelliae,* and *Kocuria salsicia* (*p* < .05). Lactose was significantly positively correlated with *Lc.piscium* and *A. pasteurianus* (*p* < .05), but negatively correlated with *E. durans* and *Entercoccus sulfurous* (*p* < .05). Most of the amino acids were significantly positively correlated with *Rothia nasimurium*, *Lb.helveticus*, *L. kefiranofaciens*, *Solibacillus silvestris,* and *G.rhaeticus* (*p* < .05), but negatively correlated with *S*. *parauberis, A. pasteurianus, M. caseolyticus*, *E. italicus,* and *E. asini* (*p* < .05).

### 3.6 The relationship between bacteria, metabolites, and nutritional composition of fermented milk

The correlation between COG functional categories and predominant bacterial species of fermented milk was analyzed to determine the role played by the bacteria in koumiss fermentation (Figure 3b). The results showed that the categories significantly correlated with *Lc.piscium* were the most abundant (*p* < .05). In addition, most functional categories were corrected with *Lactococcus lactis* and *Lb.heleveticus* (*p* < .05). Categories significantly correlated with *Lc.piscium* were the most abundant (*p* < .05). Functional categories correlated with *E. faecium*, *E. camelliae,* and *St.parauberis* were also abundant (*p* < .05). With regard to different species, there were positive correlations among *E. faecium* and functional categories A, J, R, T, I, O, Z, and D (*p* < .05). However, *E. faecium* was a negatively correlated with functional category *F* (*p* < .05). There was a negative correlation between *E. camelliae* and functional categories E, M, V, L, Q, and U (*p* < .05), and a positive correlation between *E.camelliae* and functional categories N and C (*p* < .05).

**FIGURE 4:**
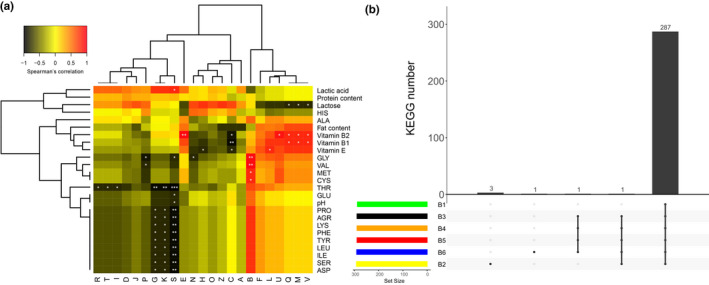
Correlation analysis between COG functional categories and koumiss active ingredients (a), and upset diagram showed the unique and shared KO content in each sample (b). Note: (a) ^*^ *p* < .05; ^**^ *p* < .01; ^***^ *p* < .001

The relationship between COG functional categories and the nutritional content of koumiss is presented in Figure 4a. There were significant negative correlations among categories G, K, and S and the amino acid content of threonine (THR), proline (PRO), arginine (ARG), lysine (LYS), phenylalanine (PHE), tyrosine (TYR), leucine (LEU), isoleucine (ILE), serine (SER), and aspartic acid (ASP). Functional category B was positively correlated with the amino acid content of glycine (GLY), valine (VAL), MET, CYS (*p* < .05). Functional categories Q, M, and V were significantly negatively correlated with lactic acid content. However, these three categories have significant positive correlation with vitamin B_1_ and vitamin B_2_.

### 3.7 Protein alignments analysis based on KEGG database at different fermentation stages

By searching and comparing all metagenomic sequences in KEGG database, a total of 7,225 different KO numbers representing different functions were identified, accounting for about 44.61% of all sequences, and a upset diagram was obtained according to the KO number of all samples (B_1_, B_2_, B_3_, B_4_, B_5_, B_6_; Figure 4b). A total of 287 KOs were shared by six different fermentation time points. There were 1–3 unique KOs at different fermentation time points.

### 3.8 Changes in metabolic pathways at different fermentation time points based on the KEGG database

**FIGURE 5:**
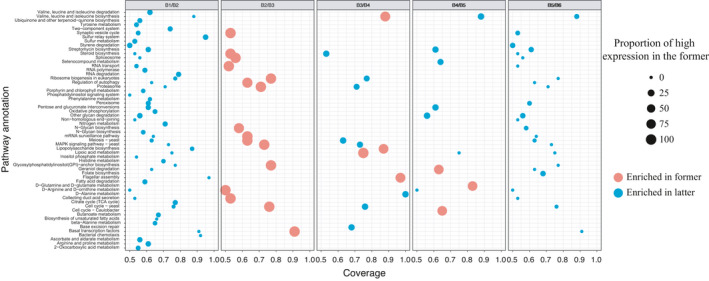
Changes in metabolic pathways at different fermentation time points based on KEGG database

There were 51 pathways that differed in activity between B_1_ and B_2_ samples using a 90% differential threshold. All of them were more active in the B_2_ sample (Figure 5). These pathways were mainly concerned with: flagellum assembly; bacterial chemotaxis; VAL, LEU, and ILE biosynthesis; glycosylphosphatidylinositol; lipoic acid metabolism; and the biosynthesis of unsaturated fatty acids. There were 16 pathways that differed in activity between the B_2_ and B_3_ samples. These pathways were also more active in the B_2_ sample. These pathways were mainly associated with: N‐glycine biosynthesis; autophagy regulation; glycosylphosphatidylinositol biosynthesis; and RNA transport. Differences between metabolic pathways activity in the B_3_ and B_4_ samples were significantly decreased. Only activity of the lipoic acid metabolic pathway was significantly higher in the B_3_ sample than in the B_4_ sample under the 90% difference threshold. However, the differentially active pathways increased to 11 when the threshold decreased to 80%. Among them, lipopolysaccharide biosynthesis, flagellum assembly, and VAL, LEU, and ILE biosynthetic pathways were more active in the B_3_ sample, and proteasome, steroid biosynthesis, and D‐alanine metabolic pathways were more active in the B_4_ samples. Differences in inactivity of metabolic pathway activity between the B_4_ and B_5_ samples were further reduced. Under the 80% threshold, only ten metabolic pathways were differentially active in the two samples. Lipoic acid metabolism, D‐arginine and ornithine D‐metabolism, selenium metabolism, streptomycin biosynthesis, pentose and glucuronic acid and conversion of VAL, LEU, and ILE biosynthesis, and other chitosan degradation pathway metabolism were more active in B_4_ samples and D‐glutamyl amine and D‐glutamate metabolism, cell cycle, and geranium campylobacter were more active in the B_5_ sample. Differences in activity may be affected by the length of the fermentation interval, and there was a significant increase in pathway activities between the B_5_ sample and the B_6_ sample. Under the 90% difference threshold, there were significant differences in 25 metabolic pathways between the B_5_ and B_6_ samples, and these 25 metabolic pathways were all more active in the B_6_ samples. The main differentially active pathways included those related to gene transcription factor, glycosylphosphatidylinositol biosynthesis, lipoic acid metabolism, geranium degradation, autophagy regulated steroid biosynthesis, transport, RNA and D‐arginine ornithine metabolism and N‐biosynthesis, GLY, VAL, LEU, and ILE biosynthesis, and the streptomycin biosynthesis pathway.

## 4 DISCUSSION

Bacterial function during fermentation of koumiss was investigated by metagenomics in the present study. *Lactococcus*, *Lactobacillus,* and *Enterococcus* were the predominant genera present during koumiss fermentation. The relative abundances of *Lactobacillus* were higher at the beginning of fermentation. These genera also play a major role in the nutritional content of koumiss. In particular, most of the amino acids were significantly positively related to *Lb.helveticus* and *Lb.kefiranofaciens*, which may be related to the role of *Lb.helveticus* in proteolysis (Sadat‐Mekmene et al., 2013). Intracellular peptidases of *Lb.helveticus* played a major role in the proteolysis of Swiss cheeses, provided that they are released through bacterial lysis (Valence et al., 1998). In this study, *Lc.piscium* was negatively correlated with fat content but positively correlated with lactose. The presence of *Lc.piscium* has also been reported in dairy products such as raw milk, using a novel multiplex PCR (Odamaki et al., 2011); and cheese, using 16S rRNA library sequencing (Carraro et al., 2011). In addition, *Enterococcus* was negatively correlated with protein. Wessels et al. (Wessels et al., 1990) and Bassem et al. (Bassem et al., 2002) also suggested that *Enterococcus* produced proteolytic enzymes involved in casein degradation. Overall, LAB played a major role in the fermentation of koumiss. Therefore, in this study is considered the role of different functional genes of LAB in different stages of fermentation from the perspective of metagenomics.

In recent years, there has been a lot of research on microbial diversity in traditionally produced yogurt. As early as 1985, Oberman and Libudzisz reported that *Lactobacillus* and *Lactococcus* were the predominant bacterial species in koumiss. It is also known that *Lb.delbrueckii* subsp. *bulgaricus* and *Lb. helveticus* have an important role in fermentation and flavor development (Oberman et al., 1985). Different researchers have isolated and identified bacteria and studied their diversity in traditional koumiss in Inner Mongolia (An et al., 2004; Ishii et al., 1997; Liu et al., 2019), Xinjiang (Sun, Liu, Zhang, Yu, Zhang, et al., 2010), and Mongolia (Danova et al., 2005; Uchida et al., 2007). In 2008, Watanabe et al. used PCR‐RAPD identification to show that *Lactobacillus* was the dominant genus in Mongolian koumiss (Watanabe et al., 2008). Xiaoqunet al. (Xiaoqun & Hesheng, 2012) studied the bacterial diversity of Xinjiang koumiss, and the results also showed that *Lactobacillus* was the dominant genus. In this study, we confirmed that *Lactobacillus* was the dominant genus in all stages of fermentation.

The functional genes of these microorganisms are bound to play an important role in the variation in type and content of metabolic products in fermented products. Thus, correlations were made between the functional genes present and nutritional content of koumiss at different stages of fermentation. Six of 23 function gene categories with high relative abundance were regarded as the major functional categories present in the six samples taken at different stages of fermentation (0, 12, 24, 36, 48, and 168 hr). Functional category L (represented replication‐, recombination‐, and repair‐related functions) was the most abundant in all of the samples. It was found that lactose content was significantly positively correlated with the secondary metabolite’ biosynthesis, transport, and catabolism. The results of the relationship between COG functional categories and nutrition in koumiss showed that categories G, K, and S were significantly positively correlated with the amino acid content of THR, PRO, ARG, LYS, PHE, TYR, LEU, ILE, SER, and ASP. From this point, we may conclude that the content of amino acids in koumiss is mainly determined by the function of bacteria related to amino acid transport and nucleic acid transport during fermentation process. We found that the functional gene categories obtained from bacterial metagenomics DNA were mostly related to LAB species in koumiss. LAB are important for decreasing pH and inhibiting spoilage bacteria. LAB are excellent candidates for preventing the growth of pathogenic bacteria in food products because they show bacteriostatic effect toward many bacterial species through various mechanisms (Ghanbari et al., 2013). Vitamin B_1_ and B_2_ were significantly correlated with secondary metabolite biosynthesis, transport and catabolism, cell wall/membrane/envelope biogenesis, and defense mechanisms. Riboflavin (VB_2_) biosynthesis has been described both in gram‐positive and gram‐negative bacteria, with detailed studies performed for *Bacillus subtilis* (Perkins et al., 2002). Fermentation process was regarded as the major way for biosynthesis of VB_2_, and a gene *rib* C had been shown to code for a bifunctional riboflavin (Neuberger & Bacher, 1986) kinase/FAD synthetase (Bacher et al., 2000).

Changes in functional gene categories at different stages of fermentation indicated in an Upset diagram. Of the functional categories, 92.7% (287) shared KOs at the four different fermentation time points which mean that there was no significant increase in KO in the first 36 hr of fermentation. The predominant bacterial species was *Lb.helveticus* between the 0 to 36 hr period of koumiss fermentation, and its relative abundance increased gradually until reaching a peak at 36 hr. Different species of *Enterococcus* also increased gradually throughout the fermentation process, reaching a maximum after 16 hr. The microbial composition of koumiss changed most before 36 hr of fermentation, and the predominant bacterial species were LAB. From this, we can infer changes in the function of microbes at different stages of fermentation might be induced by changes in the relative content of different enzymes.

The activity of particular metabolic pathways changed significantly between adjacent fermentation time points according to the bubble diagram (Figure 5). If the relative activity of a metabolic pathway at one fermentation time point was significantly higher than that at another time point, then it meant that the metabolic activity of bacteria at that time point was also more active. There were more differentially active metabolic pathways in the B_2_ sample than that in the B_1_ sample, and in the B_2_ sample compared with the B_3_ sample. Overall, significantly differentially active pathways were most abundant at the B_2_ and B_3_ fermentation points than at the other time points. These pathways included N‐glycine biosynthesis, autophagy regulation, glycosylphosphatidylinositol biosynthesis, and RNA transport. There were smaller differences of relative activity of metabolic pathway between B_4_ and B_5_ and between B_5_ and B_6_, which meant that microbial metabolism became more active during the first 24 hr of fermentation and then began to stabilize.

## CONFLICTS OF INTEREST

The authors declare that the research was conducted in the absence of any commercial or financial relationships that could be construed as a potential conflict of interest.

## ETHICAL APPROVAL

This study does not involve any human or animal testing.

## INFORMED CONSENT

Written informed consent was obtained from all study participants.
